# Neoadjuvant camrelizumab plus trastuzumab and chemotherapy for HER2-positive gastric or gastroesophageal junction adenocarcinoma: a single-arm, phase 2 trial

**DOI:** 10.1007/s10120-025-01606-w

**Published:** 2025-04-04

**Authors:** Yijie Ma, Zhi Li, Chen Wei, Jian Zhang, Qiang Fu, Zhandong Zhang, Chuang Shang, Jinbang Wang, Xiangbin Wan, Bin Zhang, Yongchao Zhang, Jing Li, He Zhang, Liangyu Bie, Qingxin Xia, Suxia Luo, Ning Li

**Affiliations:** 1 Department of Medical Oncology, The Affiliated Cancer Hospital of Zhengzhou University & Henan Cancer Hospital, 127 Dongming Road, Zhengzhou, 450000 China; 2 Department of General Surgery, The Affiliated Cancer Hospital of Zhengzhou University & Henan Cancer Hospital, Zhengzhou, 450000 China; 3 Department of Radiology, The Affiliated Cancer Hospital of Zhengzhou University & Henan Cancer Hospital, Zhengzhou, 450000 China; 4 Department of Pathology, The Affiliated Cancer Hospital of Zhengzhou University & Henan Cancer Hospital, Zhengzhou, 450000 China

**Keywords:** Gastric cancer, Gastroesophageal junction adenocarcinoma, HER2, Neoadjuvant therapy, Immunotherapy

## Abstract

**Background:**

The impact of neoadjuvant combined chemotherapy, immunotherapy, and targeted therapy on pathologic responses and survival outcomes in HER2-positive locally advanced gastric cancer remains unclear.

**Patients and Methods:**

In this single-arm, phase 2 trial, patients with HER2-positive resectable cT4 and/or N + M0 gastric or gastroesophageal junction (G/GEJ) adenocarcinoma received four cycles of neoadjuvant camrelizumab plus trastuzumab and CapOx, followed by D2 gastrectomy and four cycles of CapOx. The primary endpoint was pathological complete response (pCR, ypT0N0) rate.

**Results:**

Twenty-five patients were enrolled and received neoadjuvant combination treatment. Of these patients, 11 (44%) were in cT3 and 14 (56%) in cT4a; all had positive nodal status. Of the 23 patients who underwent surgery, 5 (21.7%, 95% CI: 7.5–43.7) achieved pCR (ypT0N0), and 7 (30.4%, 95% CI: 13.2–52.9) achieved near pCR (ypT0). The R0 resection rate was 100%. During a median follow-up of 41.0 months, no patients with pCR had recurrence or death. In contrast, five of 18 patients with non-pCR had recurrence, and four of them died. The three-year disease-free survival rate was 78.3%. During neoadjuvant treatment, grade 3 adverse events were observed in 36% of patients, with no grade 4 or 5 adverse events reported. No treatment-related surgical delay or reoperation occurred.

**Conclusion:**

Neoadjuvant camrelizumab plus trastuzumab and chemotherapy demonstrated favorable response and tolerable safety in HER2-positive G/GEJ adenocarcinoma.

**Supplementary Information:**

The online version contains supplementary material available at 10.1007/s10120-025-01606-w.

## Introduction

Approximately 15–20% of gastric cancer patients overexpress human epidermal growth factor receptor-2 (HER2) [1]. Currently, the standard treatment for HER2-positive resectable gastric or gastro-esophageal junction (G/GEJ) adenocarcinoma is radical surgery with perioperative chemotherapy [2]. However, treatment outcomes remain unsatisfactory, with a two-year disease-free survival (DFS) rate of only 54% [3]. New therapies for patients with HER2-positive resectable G/GEJ adenocarcinoma are crucially needed.

Trastuzumab is the first HER2-targeted agent recommended for first-line treatment of HER2-positive metastatic gastric cancer, based on the results of the pivotal TOGA trial [4]. It has also shown antitumor activity in locally advanced disease when combined with neoadjuvant chemotherapy [5, 6]. Hower, the observed antitumor activity has been suboptimal, with reported pathologic complete response (pCR, ypT0N0) rates of 14.3%, and near pCR (ypT0) rates ranging from 9.6 to 21.4% [5, 7, 8]. Resistance to trastuzumab in this setting may be related to the interaction between HER2 overexpression and increased levels of programmed death-ligand 1 (PD-L1) [9]. Therefore, combining trastuzumab with programmed death 1 (PD-1) inhibitors may further enhance antitumor efficacy due to their potential synergistic effects [10]. Notably, based on the findings of the KEYNOTE-811 study, the FDA has approved pembrolizumab plus trastuzumab and chemotherapy as a first-line treatment for HER2-positive locally advanced or metastatic G/GEJ adenocarcinoma [11]. These developments provide a rationale for further investigation of this combination in the neoadjuvant setting for HER2-positive resectable G/GEJ adenocarcinoma.

Preliminary reports from recent phase 2 trials, presented at the 2023 European Society for Medical Oncology (ESMO) and 2024 American Society of Clinical Oncology (ASCO) meetings have shown encouraging pCR rates in patients with HER2-positive resectable G/GEJ adenocarcinoma treated with PD-1 inhibitors plus trastuzumab and chemotherapy [7, 12]. Specifically, perioperative treatment with atezolizumab combined with trastuzumab and CapOx (oxaliplatin, capecitabine) resulted in a pCR (ypT0N0) rate of 38.1%, which is significantly higher than 14.3% observed in the trastuzumab plus CapOx group [7]. Additionally, perioperative treatment with tislelizumab plus trastuzumab, docetaxel and oxaliplatin showed a near pCR (ypT0) rate of 42.9% in seven patients who underwent surgery [12]. These trials are ongoing, and final outcomes are awaited to confirm the durability of these responses.

Camrelizumab, another PD-1 inhibitor, has been approved as a first-line treatment for advanced esophageal cancer and several other solid tumors. In combination with trastuzumab and chemotherapy, camrelizumab has shown superior efficacy compared to trastuzumab plus chemotherapy alone in the treatment of HER2-positive metastatic gastric cancer [13, 14]. In light of these findings, this study aimed to evaluate the antitumor activity and safety of camrelizumab combined with trastuzumab and CapOx as neoadjuvant therapy for patients with HER2-positive G/GEJ adenocarcinoma.

## Methods

### Study design and participants

This single-arm, phase 2 trial was conducted at Henan Cancer Hospital. The trial was approved by the Ethics Committee of Henan Cancer Hospital (NO. 2019211) and conducted in adherence with the Declaration of Helsinki and Good Clinical Practice guidelines. Written informed consent was obtained from all patients before enrollment. The trial was registered at ClinicalTrials.gov with the identifier NCT03950271.

Eligible patients were aged 18–75 years; had pathologically confirmed G/GEJ adenocarcinoma with cT4 and/or N + M0 per American Joint Committee on Cancer (AJCC) staging manual 8th edition; resectable as determined by computed tomography (CT) or magnetic resonance imaging (MRI) for distant metastases, bone scan for suspected bone metastasis, laparoscopy for suspected peritoneal metastasis and CT or MRI for suspected brain metastasis; CT confirmed absence of peritoneal metastasis; a multidisciplinary team (MDT) believed that perioperative treatment was needed; HER2 positive status in tumors (immunohistochemistry (IHC) 3 + or IHC 2 + with fluorescence in situ hybridization positivity); an Eastern Cooperative Oncology Group performance status of 0–1; and no prior chemotherapy, targeted therapy, or local tumor resection. Exclusion criteria included prior immunotherapies (anti-CTLA4, anti-PD-1, or anti-PD-L1 antibodies); and a history of or active autoimmune diseases.

### Procedures

Eligible patients received four cycles of neoadjuvant therapy with camrelizumab (200 mg, iv, day 1, q3w), trastuzumab (initially 8 mg/kg, followed by 6 mg/kg, iv, day 1, q3w) and CapOx (oxaliplatin 130 mg/m^2^, iv, day 2, and capecitabine 1000 mg/m^2^, orally bid, days 1–14, q3w). When this trial was designed, no data was available on the appropriate interval between the last dose of camrelizumab and surgery. Thus, the administration of the fourth cycle of camrelizumab was at the investigator’s discretion to minimize safety concerns. Before surgery, the MDT reassessed the clinical stage and the resectability of the tumor. Patients deemed resectable underwent gastrectomy and D2 lymphadenectomy within two to six weeks following neoadjuvant therapy. Those who prematurely discontinued neoadjuvant therapy, but were still considered resectable, proceeded to surgery. Adjuvant therapy with four cycles of CapOx started within three to eight weeks after surgery. Treatment was continued until the completion of the prescribed cycles, disease progression, unacceptable toxicity, or consent withdrawal.

Imaging assessments were performed by CT or MRI every six weeks during neoadjuvant and adjuvant treatment, then every three months for up to three years, and every six to eight months for up to five years. Gastroscopy was repeated annually. Survival status was followed up every three months for the first three years and every six months thereafter. Radiologic response was evaluated based on RECIST v1.1 criteria. Pathologic response, including ypTxNxMx, tumor regression grade (TRG), and R0 resection, were evaluated based on the AJCC 8th edition. TRG 0, no viable cancer cells (complete response); TRG 1, single cells or rare small clusters of cancer cells (near-complete response); TRG 2, residual cancer cells with obvious tumor regression, but more than single cells or rare small groups of cancer (partial response); TRG 3, substantial residual cancer with no obvious tumor regression (poor or no response). Adverse events (AEs) were graded based on the National Cancer Institute Common Terminology Criteria for Adverse Events version 5.0. Surgical complications were assessed based on the Clavien-Dindo classification.

### Outcomes

The primary endpoint was pCR (ypT0N0) rate, defined as the proportion of patients with no residual tumor cells in both the resected tumor tissue and lymph nodes. Secondary endpoints included near pCR (ypT0) rate (defined as the proportion of patients with no residual tumor cells in the resected tumor tissue), TRG rate, R0 resection, downstaging, intraoperative blood loss, operative duration, postoperative hospital stay, postoperative 90-day mortality, secondary surgery, objective response rate (ORR), disease-free survival (DFS, defined as the duration from surgical procedure to the first occurrence of disease recurrence or death), event-free survival (EFS, defined as the duration from the initiation of neoadjuvant treatment to the first occurrence of disease recurrence or death), overall survival (OS, defined as the duration from the initiation of neoadjuvant treatment to death from any cause) and safety.

### Statistical analysis

The pCR (ypT0N0) rate of neoadjuvant chemotherapy was 5% [15]. Assuming neoadjuvant camrelizumab plus trastuzumab, oxaliplatin and capecitabine could achieve a pCR rate of 20%, a sample size of 22 would provide at least 80% power at an overall one-sided *α* = 0.05 to demonstrate the efficacy of the test neoadjuvant treatment was promising. Considering 10% drop-out, 25 patients were required to be enrolled.

All patients who received at least one dose of the study drug were included in the full analysis set (FAS) and safety analysis set (SAS). The surgery set (SS) comprised all patients who underwent gastrectomy. Efficacy endpoints were analyzed in FAS and SS. Neoadjuvant safety and surgical complications were evaluated in SAS and SS, respectively. Adjuvant safety was evaluated in patients who received at least one dose of CapOx during adjuvant phase. Continuous data were described as median (range), and categorical data were described as number (percentage). The pCR rate, near pCR rate, TRG rate, and ORR were calculated and the corresponding 95% confidence intervals (CI) were calculated by the Clopper-Pearson method. The median and two-year or three-year probability of DFS, EFS, and OS were estimated by the Kaplan–Meier method, and the 95% CI of two/three-year survival probability were calculated using Greenwood's formula. Subgroups analysis of survival outcomes were grouped by pathological responses (pCR *vs.* non-pCR). Statistical analyses were executed with SAS software (version 9.4).

## Results

### Patients and treatment

**Fig. 1:**
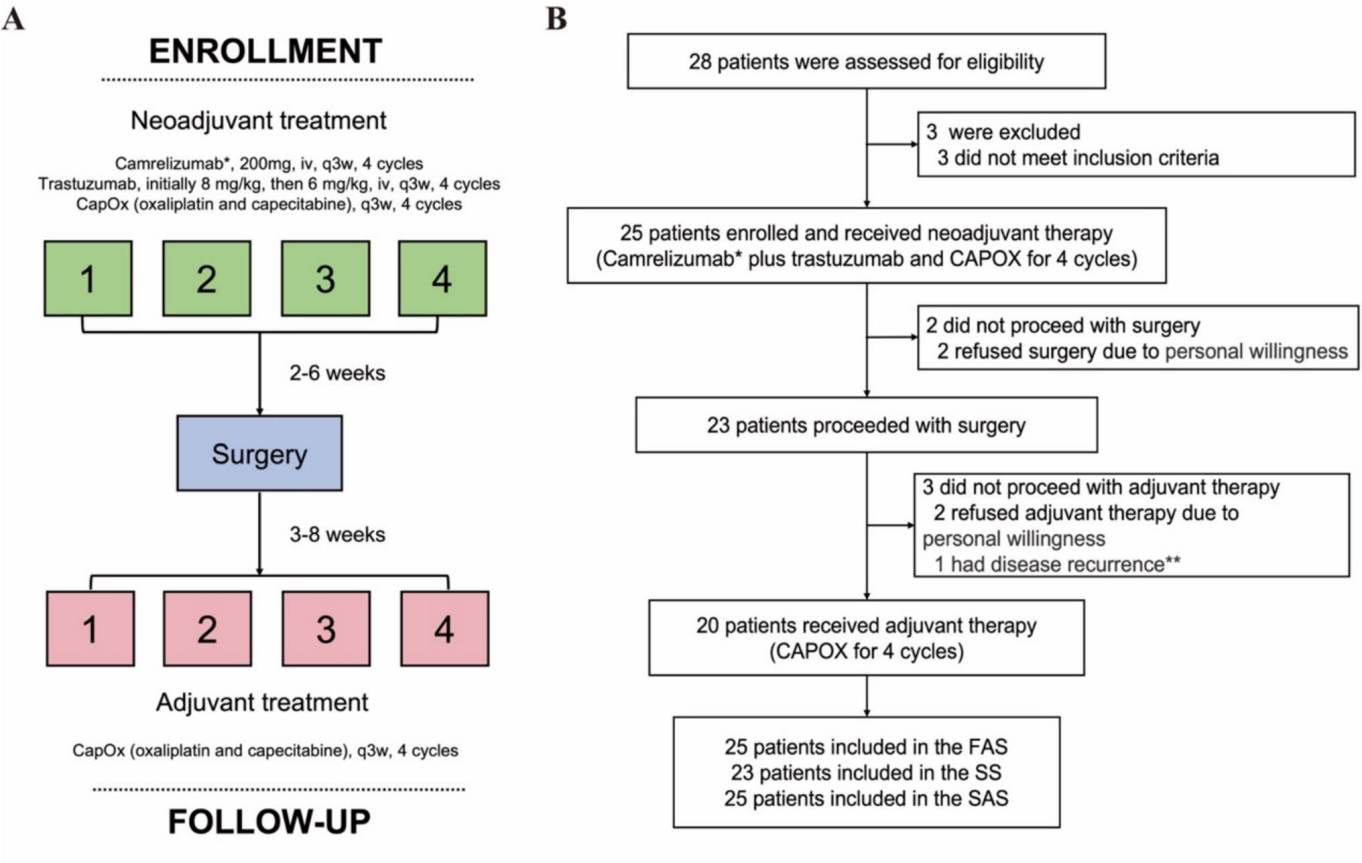
Treatment schedule and patient flow chart. CapOx, oxaliplatin plus capecitabine; FAS, full analysis set; SS, surgery set; SAS, safety analysis set. ^*^ The administration of the fourth cycle of camrelizumab was at the investigator’s discretion to minimize safety concerns. ^** ^This relapsed patient had severe disease with baseline staging of T4aN2, and achieved ypT3N2 and TRG3 after neoadjuvant treatment

From January 2020 to January 2022, 25 eligible patients were enrolled and all received neoadjuvant treatment with camrelizumab plus trastuzumab and chemotherapy (Fig. 1). The median age was 61 years (range 50–75) and 20 patients (80.0%) were male. Eighteen (72.0%) patients had primary gastric cancer. All patients had stage III disease, including 14 (56.0%) at cT4a and 25 (100%) having lymph node involvement. Of these patients, 11 (44.0%) had PD-L1 CPS of ≥ 1, and 24 (96.0%) had microsatellite stable status (Table 1). The microsatellite status for one patient was unavailable.

**Table 1:** Baseline demographics and disease characteristics (n = 25)

Characteristics	Patients
Age, years	61 (50–75)
Male	20 (80.0)
Primary tumor location	
Gastric	18 (72.0)
Gastric-esophageal junction	7 (28.0)
Histologic grade	
Highly differentiated	1 (4.0)
Moderately differentiated	10 (40.0)
Poorly differentiated	5 (20.0)
Grade cannot be assessed	9 (36.0)
Clinical tumor stage	
cT3	11 (44.0)
cT4a	14 (56.0)
Clinical node stage	
cN +	25 (100.0)
cN1	4 (16.0)
cN2	13 (52.0)
cN3	8 (32.0)
Clinical disease stage	
III	25 (100.0)
Microsatellite status	
MSS	24 (96.0)
Unavailable	1 (4.0)
PD-L1 (CPS)	
< 1	14 (56.0)
≥ 1	11 (44.0)

Data are median (range) or n (%)

*MSS* microsatellite stable, *PD-L1* programmed death-ligand 1, *CPS* combined positive score

Treatment details, including the number of cycles for each regimen, are shown in Supplementary Table 1. Of the 25 patients, 24 (96%) completed three cycles of camrelizumab, 18 (72.0%) completed four cycles neoadjuvant trastuzumab plus CapOx, and three (12.0%) opted for early surgery after three cycles neoadjuvant therapy. Following neoadjuvant treatment, two patients declined surgery due to personal willingness, leading to 23 patients proceeding with surgery. Postoperatively, 20 (87.0%) of these 23 patients received adjuvant CapOx, with 14 (70.0%) completing all prescribed cycles.

### Antitumor responses

**Fig. 2:**
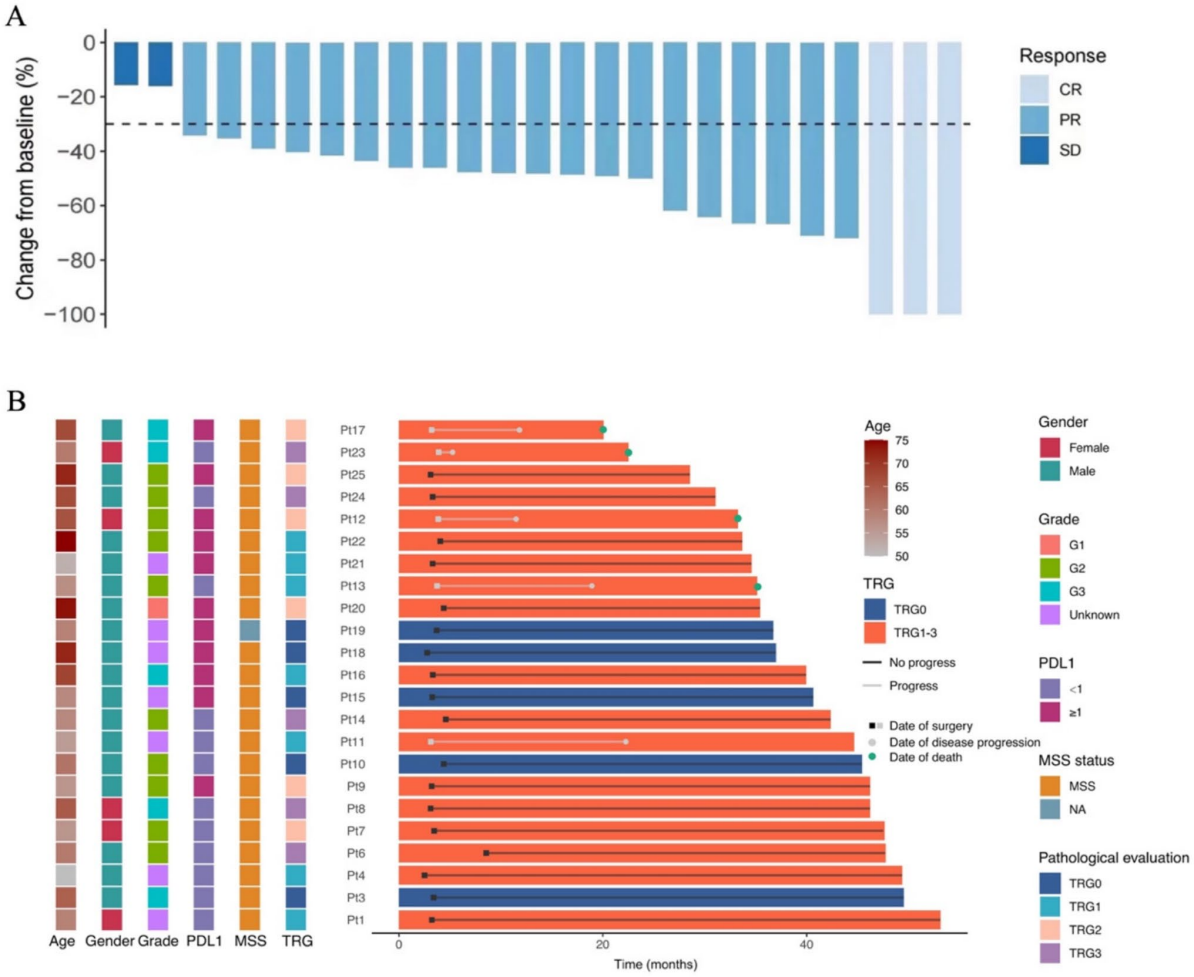
Radiological and pathological responses. **a**. Waterfall plot of tumor size change from baseline to maximum percentage (n = 25) **b**. Swimming plot of events during follow-up (n = 23). Radiological tumor response was assessed based on RECIST v 1.1. The pathological response was assessed according to the AJCC 8th edition. CR, complete response; PR, partial response; SD, stable disease. PD-L1, programmed death-ligand 1; MSI, microsatellite instability; MSS, microsatellite stable; TRG, tumor regression grade; Pt, patient

All 25 patients were evaluable for radiological response. The objective response rate (ORR) per RECIST v1.1 was 92.0% (95%CI: 74.0–99.0), with 3 complete response (CR) and 20 partial response (PR) (Table 2). No patients experienced progressive disease (PD) during neoadjuvant treatment (Fig. 2).

**Table 2:** Radiological response in the FAS and pathological response in the surgery set

	Patients
Radiological response	Patients (n = 25)
CR	3 (12)
PR	20 (80)
SD	2 (8)
PD	0
ORR	23 (92.0, 95%CI: 74.0–99.0)
DCR	25 (100, 95%CI: 86.3–100)
Pathological response	Patients (n = 23)
Pathological T stage (ypT)	
T0	7 (30.4)
T1	4 (17.4)
T2	2 (8.7)
T3	10 (43.5)
Downing T	19 (82.6)
Pathological nodal stage (ypN)	
N0	14 (60.9)
N1	5 (21.7)
N2	3 (13.0)
N3	1 (4.3)
Downing N	19 (82.6)
Nerve invasion	
0	8 (34.8)
1	6 (26.1)
Unknown	9 (39.1)
Vessel invasion	
0	10 (43.5)
1	4 (17.4)
Unknown	9 (39.1)
R0 resection	23 (100.0)
pCR (ypT0N0)	5 (21.7, 95%CI: 7.5–43.7)
Near pCR (ypT0)	7 (30.4, 95%CI:13.2–52.9)
TRG	
TRG0	5 (21.7, 95%CI:7.5–43.7)
TRG1	7 (30.4, 95%CI:13.2–52.9)
TRG2	6 (26.1, 95%CI:10.2–48.4)
TRG3	5 (21.7, 95%CI:7.5–43.7)
TNM stage (post-neoadjuvant)	
0	5 (21.7)
I	8 (34.8)
II	6 (26.1)
III	4 (17.4)

Data are median (range) or n (%, 95% CI). TRG was assessed according to the American Joint Committee on Cancer staging manual 8th edition. FAS, full analysis set; pCR, pathological complete response; TRG, tumor regression grade

Twenty-three patients who underwent surgery were evaluable for pathological response (Fig. 2). Of these, the R0 resection rate was 100%. Downstaging of the primary tumor, nodal status, and TNM classification was each observed in 82.6% of patients (Table 2). The pathological complete response (pCR, ypT0N0) rate was 21.7% (95% CI: 7.5–43.7), and the near pCR (ypT0) rate was 30.4% (95% CI: 13.2–52.9) (Table 2). The pCR rate for all 25 patients was 20% (95% CI: 8.9–39.1). Subgroup results of pathological response are shown in Supplementary Table 2. The pCR rates in PD-L1-positive tumors (CPS ≥ 1) and PD-L1negative (CPS < 1) tumors were 27.3% (3/11) and 16.7% (2/12), respectively.

The rates of TRG0 (equal to ypT0N0), TRG1 and TRG2 were 21.7% (95%CI: 7.5–43.7), 30.4% (95% CI: 13.2–52.9), and 26.1% (95% CI: 10.2–48.4), respectively. The combined TRG0/1 rate was 52.2% (Table 2).

### Survival outcomes

**Fig. 3:**
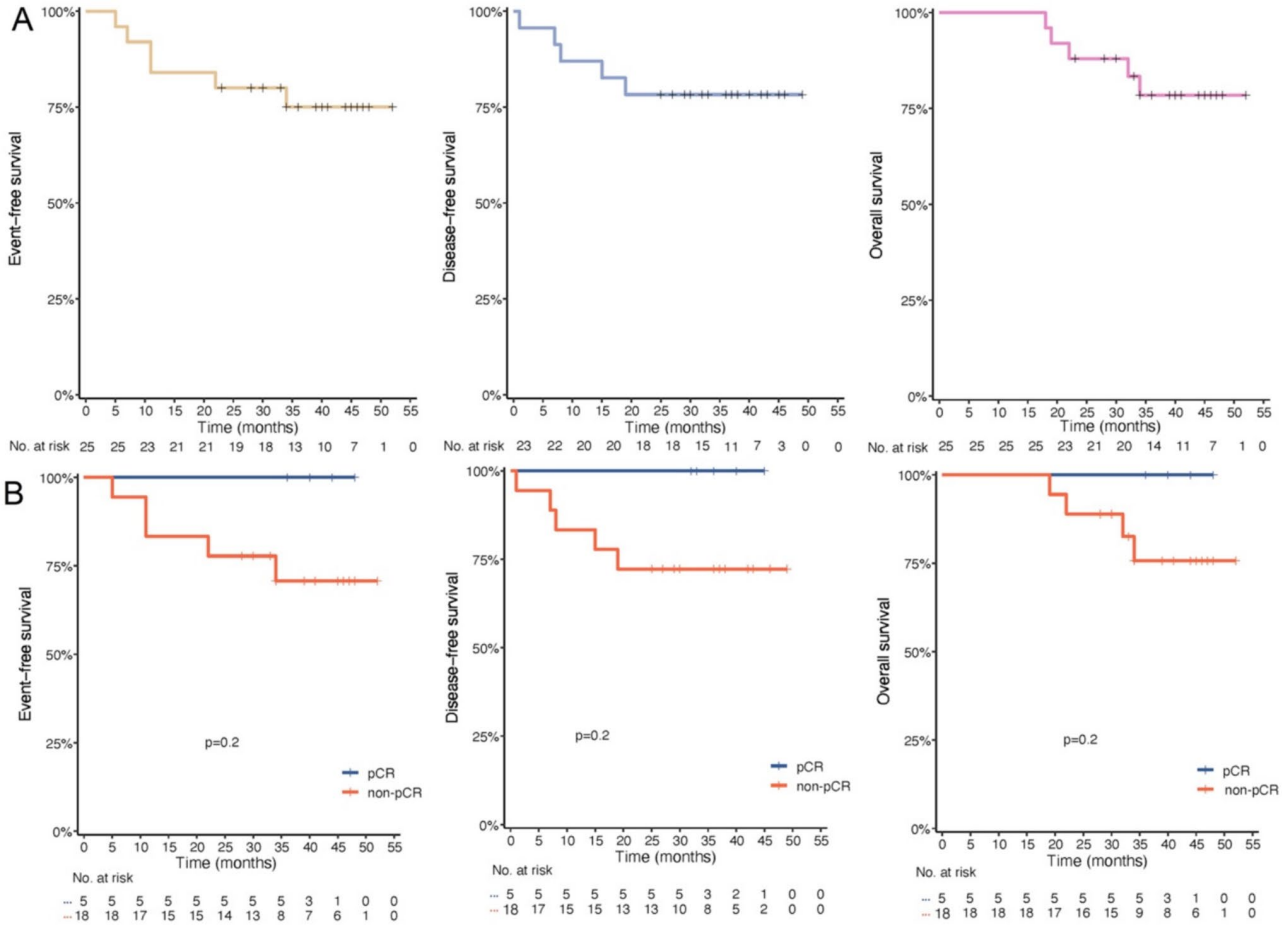
Survival outcomes. **A**. Kaplan–Meier curves for EFS and OS in the FAS (n = 25), and DFS in the surgery set (n = 23). **B**. Kaplan–Meier curves for EFS, DFS and OS in the surgery set (n = 23) between pCR and non-pCR. EFS, event-free survival; OS, overall survival; DFS, disease-free survival; FAS, full analysis set; pCR, pathologic complete response

As of the data cutoff on June 1, 2024, the median follow-up was 41.0 months (range: 36–46). Median DFS, EFS, and OS were not reached. The two-year DFS, EFS, and OS rates were 78.3% (95% CI: 63.1–97.1), 80.0% (95% CI: 65.8–97.3) and 88.0% (95% CI: 76.1–100), respectively. The three-year DFS, EFS, and OS rates were 78.3% (95% CI: 63.1–97.1), 75.0% (95% CI: 59.4–94.7), and 78.5% (95% CI: 63.3–97.3), respectively (Fig. 3). Of the five patients who achieved pCR (ypT0N0), all had no recurrence or death. Of the 18 patients who achieved non-pCR, five had recurrence and four of them died (Fig. 3). The three-year survival rates of DFS (100% vs. 72.2%), EFS (100% vs. 72.2%) and OS (100% vs. 75.7%) were numerically higher in patients with pCR compared with those with non-pCR (Fig. 3).

### Safety and feasibility

During neoadjuvant treatment, any-grade treatment-emergent adverse events (TEAEs) occurred in all patients, but grade 3 TEAEs occurred only in 9 (36%) of 25 patients (Table 3). The most common any-grade TEAEs were nausea (21 [84.0%]), fatigue (21 [84.0%]), and appetite decreased (21 [84.0%]). No grade 4 or 5 TEAEs were reported. No TEAEs led to a discontinuation of neoadjuvant treatment. A complete list of the immune-related adverse events is shown in Supplementary Table 4.

**Table 3:** Treatment-emergent adverse events during neoadjuvant treatment (n = 25)

TEAEs	Any grade	Grade 1	Grade 2	Grade 3
Hematological				
Lymphopenia	20 (80.0)	8 (32.0)	10 (40.0)	2 (8.0)
Anemia	18 (72.0)	9 (36.0)	7 (28.0)	2 (8.0)
Platelet count decreased	15 (60.0)	9 (36.0)	3 (12.0)	3 (12.0)
Neutropenia	12 (48.0)	4 (16.0)	6 (24.0)	2 (8.0)
Leukopenia	10 (40.0)	3 (12.0)	7 (28.0)	0
Non-hematological				
Nausea	21 (84.0)	18 (72.0)	3 (12.0)	0
Fatigue	21 (84.0)	20 (80.0)	1 (4.0)	0
Appetite decreased	21 (84.0)	20 (80.0)	1 (4.0)	0
Vomiting	20 (80.0)	19 (76.0)	1 (4.0)	0
Hypoproteinemia	20 (80.0)	18 (72.0)	2 (8.0)	0
RCCEP	10 (40.0)	9 (36.0)	1 (4.0)	0
AST increased	10 (40.0)	9 (36.0)	1 (4.0)	0
Peripheral neuropathy	9 (36.0)	9 (36.0)	0	0
ALT increased	9 (36.0)	9 (36.0)	0	0
CPK increased	8 (32.0)	7 (28.0)	0	1 (4.0)
Direct bilirubin increased	8 (32.0)	7 (28.0)	1 (4.0)	0
Indirect bilirubin increased	5 (20.0)	5 (20.0)	0	0
Diarrhea	4 (16.0)	2 (8.0)	1 (4.0)	1 (4.0)
Increased creatinine	4 (16.0)	4 (16.0)	0	0
Hyperbilirubinemia (total bilirubin)	3 (12.0)	3 (12.0)	0	0
Palmar-plantar erythrodysesthesia	2 (8.0)	2 (8.0)	0	0
Pruritus	2 (8.0)	2 (8.0)	0	0
Pneumonia	1 (4.0)	0	1 (4.0)	0
Hypothyroidism	2 (8.0)	1 (4.0)	1 (4.0)	0
ALP increased	1 (4.0)	0	1 (4.0)	0
Abdominal pain	1 (4.0)	1 (4.0)	0	0
Hyperthyroidism	1 (4.0)	1 (4.0)	0	0

Data are n (%). No ≥ grade 4 TEAEs were observed

*TEAEs* treatment-emergent adverse events, *ALP* alkaline phosphatase, *ALT* alanine aminotransferase increase, *AST* aspartate aminotransferase, *CPK* creatine phosphokinase, *RCCEP* reactive cutaneous capillary endothelial proliferation

The median interval from the last neoadjuvant treatment to surgery was 36.3 days (range: 24–85). There were no treatment-related delays or terminations of surgery. The number of lymph nodes cleared during surgery was 26.9 (range: 14–46). Details of the blood loss, duration of operation, and median hospital stay are provided in Supplementary Table 3. There were no surgery-related death or reoperation within 90 days after surgery. 20 (87.0%) of 23 patients experienced surgical complications, all of which were grade 1 or 2. The most common surgical complication was nausea (21 [84.0%]) (Supplementary Table 5). One patient experienced chylous leakage and fully recovered after symptomatic treatment.

Post-surgery, the median time to start adjuvant CapOx was 32.2 days (range: 24–47). During adjuvant CapOx, 8 (40.0%) of 20 patients had grade ≥ 3 TEAEs, with no unexpected TEAEs reported (Supplementary Table 6).

### Exploratory biomarkers

Peripheral blood immune cells were collected from patients before neoadjuvant therapy and post-surgery. At baseline, significant differences were observed in the percentages of CD3 + CD8 + T cells, CD16 + CD56 + NK cells, CD19 + B cells and the absolute counts of CD19 + B cells between the TRG0 (marked tumor regression) and non-TRG0 (TRG1-3, mild or no regression) groups (Supplementary Fig. 1). After surgery, PD-1 + CD4 + T cells significantly decreased in both the TRG0 and non-TRG0 groups compared to baseline levels (Supplementary Fig. 2).

## Discussion

We reported that the neoadjuvant combination of camrelizumab, trastuzumab, and chemotherapy had notable antitumor activity in HER2-positive resectable G/GEJ adenocarcinoma. This regimen achieved a pCR (ypT0N0) rate of 21.7% and a near pCR (ypT0) rate of 30.4%, with a R0 resection rate of 100%. During a median follow-up of 41.0 months, no recurrence or death occurred in patients who achieved pCR. And the three-year DFS, EFS, and OS rates were 78.3%, 75.0%, and 78.5%, respectively. In addition, this regimen was safe and feasible, with no treatment-related surgical delay or unexpected AEs reported. These findings provide new data supporting the integration of immunotherapy into the neoadjuvant treatment of HER2-positive G/GEJ adenocarcinoma. To our knowledge, this is the first report detailing both pathologic response and long-term survival outcomes for neoadjuvant immunotherapy combined with HER2-targeted therapy and chemotherapy in G/GEJ adenocarcinoma.

The optimal regimen for neoadjuvant treatment of HER2-positive gastric cancer is still under investigation. Several previous phase II trials have evaluated the efficacy of targeted therapy combined with chemotherapy or a combination of targeted therapy, chemotherapy and immunotherapy. Both trastuzumab combined with chemotherapy and the combination of trastuzumab and pertuzumab with chemotherapy have been shown to enhance the pCR rate. For example, the NEOHX trial reported a near pCR rate of 9.6% for trastuzumab combined with CapOx, while the HER-FLOT trial demonstrated a near pCR rate of 21.4% for trastuzumab combined with FLOT [5, 8]. Our findings, in conjunction with existing preclinical and clinical data, support the favorable effects of adding PD-1 inhibitors to this treatment approach. Preclinical studies suggest that combining anti-HER2 agents with PD-1 inhibitors yields synergistic antitumor effects [9, 16, 17]. Clinically, the addition of PD-1 inhibitors to chemotherapy has been shown to improve pathological responses in resectable gastric cancer [18, 19].

In addition, the results of studies investigating the combination of immunotherapy, HER2-targeted therapy, and chemotherapy in HER2-positive resectable G/GEJ adenocarcinoma are still preliminary due to incomplete patient enrollment. At the ASCO 2024 meeting, a trial evaluating atezolizumab combined with trastuzumab and CapOx reported a pCR rate of 38.1% [7]. Another trial presented at the ESMO 2023 meeting demonstrated a near pCR rate of 42.9% for tislelizumab combined with trastuzumab and chemotherapy in 7 patients who underwent surgery [12]. In contrast, the pCR rate in this trial appears to be lower, possibly for several reasons. This study included patients with more advanced disease (all were N + , cT3–T4, and stage III), whereas only 50% of patients had N + and 25% had cT2 in the tislelizumab trial, and 9.5% of those in the atezolizumab trial had stage II disease [7, 12]. Additionally, the proportion of patients with PD-L1 positivity, a known predictor of better immunotherapy response in gastric cancer, was lower in this study (56% vs. 71.4% in the atezolizumab trial) [7, 20]. Differences in chemotherapy regimens may also contribute. This study used CapOX, whereas the tislelizumab trial used DOS [12]. Previous data suggest that DOS may be more effective than CapOX in the neoadjuvant treatment of gastric cancer [21]. As the final and complete data from these two trials have not yet been disclosed, the above direct comparisons should be treated with caution. Overall, the existing findings collectively suggest that integrating immunotherapy with HER2-targeted therapy and chemotherapy is a promising approach for improving treatment outcomes in patients with HER2-positive G/GEJ adenocarcinoma.

The AE profile for neoadjuvant camrelizumab plus trastuzumab and CapOx was similar with previously reported safety data [5, 22]. No new safety signals were identified. Grade ≥ 3 TEAEs occurred in only 36% of patients, all of which were fully recovered with management. There was no report of severe or life-threatening immune-related AEs, such as myocarditis or pneumonitis. No AE-related surgery delay or termination were reported. All patients who underwent surgery achieved R0 resection, with no secondary surgery or death within 90 days postoperatively. Additionally, there were no unexpected surgical and postoperative complications compared with previous studies [3, 15]. No AE-related deaths occurred during the entire perioperative treatment.

This study has several limitations. First, the single-arm design and the constrained sample size preclude the determination of individual efficacy contributions from camrelizumab, trastuzumab, and CapOx. Future randomized controlled trials are necessary to validate our preliminary findings. Second, this study lacked a comprehensive biomarker analysis to identify the optimal beneficial population. Thus, further investigation is necessary to clarify who benefits most from our treatment regimen.

In conclusion, neoadjuvant therapy with camrelizumab, trastuzumab, and chemotherapy demonstrates promising antitumor response and acceptable safety profile in HER2-positive resectable G/GEJ adenocarcinoma. These findings may provide a potentially subtype-specific treatment option for this population and warrants exploration in future clinical trials.

## Supplementary Information

Below is the link to the electronic supplementary material.

Supplementary file1 (DOCX 1894 KB)

## Data Availability

The data collected in this study will be available upon request to the corresponding author Ning Li (lining97@126.com).
