# Supplementary material for: Neoadjuvant camrelizumab plus trastuzumab and chemotherapy for HER2-positive gastric or gastroesophageal junction adenocarcinoma: a single-arm, phase 2 trial
Source: Gastric Cancer. 2025 Apr 4;28(4):652–61. doi: 10.1007/s10120-025-01606-w (PMC12174190; doi:10.1007/s10120-025-01606-w)
Supplement: Supplementary file 1 — Supplementary file1 (DOCX 1894 KB) [file 10120_2025_1606_MOESM1_ESM.docx]

Supplementary Materials for

**Neoadjuvant camrelizumab plus trastuzumab and chemotherapy for HER2-positive gastric or gastroesophageal junction adenocarcinoma: a single-arm, phase 2 trial**

*Corresponding author:

Prof. Ning Li, Email: lining97@126.com

**This PDF file includes:**

Supplementary methods

Figs. S1 to S2

Tables S1 to S6

# Supplementary methods

**Immunohistochemical (IHC) analysis of mismatch repair (MMR) protein expression**

Immunohistochemical analysis (IHC) was performed on 4 µm thick whole sections of FFPE tumor tissue for each case. Mismatch repair (MMR)-IHC was carried out using four antibodies directed against MLH1 (M1 Ventana clone ready for use and Optiview kit revelation, Tucson, AZ, USA), MSH2 (clone G219-1129 Ventana ready to use; Optiview kit revelation), MSH6 (clone SP93 Ventana ready to use; Optiview kit revelation), and PMS2 (clone A16-4 Ventana ready to use; Optiview kit revelation) proteins on the Ventana Benchmark XT autostainer (Ventana Medical Systems Inc., Tucson, AZ). Adjacent normal tissue from each sample served as positive controls. MMR protein loss was defined by the absence of IHC staining in the nucleus of tumor cells while normal cells remained stained, ensuring the technical validity of the experiment. IHC staining results were evaluated according to the scoring system reported in the literature: (i) proficient MMR (pMMR), cases showing positive staining of all four MMR; (ii) defective expression of mismatch repair proteins (dMMR), cases carrying the loss of one of two heterodimers, including MLH1/PMS2 or MSH2/MSH6 loss [9]. We further considered another subset: (iii) cases harboring the loss of one MMR and/or the patchy expression of one or more MMR (lo-paMMR). Two independent observers carried out the immunohistochemical analysis, and both were blinded.

**Next-generation sequencing (NGS) detection for microsatellite status**

Paired tumor and blood tissue DNA samples were extracted from FFPE samples and sequenced using Illumina TruSightTM Oncology 500 (TSO500) for MSI status determination. The library was prepared according to the manufacturer’s protocol using a hybrid capture-based TruSight Oncology 500 DNA/RNA NextSeq Kit (Illumina, San Diego, CA, USA). During the library preparation, enrichment chemistry was optimized to capture nucleic acid targets from FFPE tissues. Barcoded libraries were hybridized into a multiple-gene panel covering whole exons and selected introns of MMR-related genes, including MLH1, MSH2, MSH6, PMS2, and EPCAM. These libraries were sequenced on an Illumina NextSeq 500 platform and assessed for variants including single nucleotide variants, small insertions and deletions (indels), copy number alterations, and gene fusions/rearrangements. In the TSO 500 analysis, unique molecular identifiers were used to determine the unique coverage at each position and to reduce the background noise caused by sequencing and deamination artifacts in the FFPE samples. The MSI score was calculated using 130 homopolymer microsatellite loci targeted by the TSO500 panel according to the manufacturer’s instructions. The proportion of unstable MSI sites to the total assessed MSI sites was reported as a sample-level microsatellite score, in which at least 40 sites were required to determine an MSI score. The MSI status was calculated from microsatellite sites for evidence of instability relative to a set of baseline normal samples that are based on information entropy metrics. The final NGS results were reviewed by a bioinformatics expert.

**Immunohistochemical (IHC) analysis of PD-L1 protein expression**

For evaluating PD-L1 expression based on the 22C3-IHC assay, the CPS was calculated by dividing the number of stained cells expressing PD-L1 (tumor cells, lymphocytes, and macrophages) with the total number of viable tumor cells, and by multiplying the quotient by 100. CPS ≥ 1 was considered as positive.

**Immunohistochemical (IHC) analysis of HER-2 protein expression**

Tissue preparation and immunohistochemical staining methods were as follows. The 4-μm sections of FFPE tumor blocks freshly cut from GC patients were subjected to heat-induced antigen retrieval, and then prepared for HER2 IHC assays. To control costs, two paraffin blocks from one patient were sliced onto one slide for all assays. HER2 assessment was performed on the whole sections of FFPE tumor blocks of surgical specimens, utilizing the anti-HER2 (4B5) rabbit monoclonal antibody. IHC staining was conducted with the iView DAB Detection Kit on a BenchMark XT automated stainer following the manufacturer's procedures. Negative controls (without primary anti-body) and positive controls (GC with known HER2 IHC3+) were included in each staining run. All the reagents and instruments were obtained from Ventana Medical Systems, Tucson, AZ, USA.

HER2 evaluation and heterogeneity assessment methods were as follows:

HER2 IHC staining was assessed on the basis of the established criteria for surgical samples, that is, no or less than 10% tumor cell positive staining was the score of 0; faint or barely perceptible staining on at least 10 % tumor cell membrane was the score of 1+; weak to moderate complete or basolateral staining on at least 10% tumor cell membrane was 2+; moderate to strong complete or basolateral staining on at least 10% tumor cell membrane was 3+. Scores of 0/1+ were considered to be HER2 negative, and 2+/3+ were considered to be HER2 overexpression, in which 3+ was defined as positive and 2+ as equivocal. Two independent gastrointestinal pathologists evaluated the HER2 status without any clinical information. When disagreement occurred, a third pathologist was further consulted, and the discrepancies were resolved by consensus.

**FISH analysis of HER-2 amplification**

The FISH test was performed on FFPE slides with a thick-ness of 4-5 μm using PathVysion HER2 DNA probe kit (Vysis/Abbott, Abbott Park, Illinois) based on the ThermoBrite Elite automated FISH slides prep system (Leica, Richmond, CA, USA) according to the instruction manual. HER2 and CEP 17 signals were counted in at least 20 cell nuclei from at least two areas of invasive tumor under the CytoVision DM6000B fluorescent microscope system (Leica, Biosystem, Buffalo Grove, IL). The interpretation criteria of FISH signals for positive, negative, and equivocation were recognized as follows: a ratio of HER2/CEP17≥2.0 or an average of HER2 signals/cell ≥ 6.0 with a HER2/CEP17 ratio of < 2.0; a ratio of HER2/CEP17 < 2.0 with an average of HER2 signals/cell < 4.0; and an average of HER2 signals/cell ≥ 4.0 and < 6.0 HER2 with a HER2/CEP17 ratio of < 2.0, respectively, according to the 2013 ASCO/CAP HER2 test guidelines.

**Flow cytometry**

Whole blood samples were collected from patients before neoadjuvant therapy and post-surgery. Peripheral blood mononuclear cells (PBMCs) were isolated, and the peripheral blood immune cells (PBICs) were detected by flow cytometry. The cell surfaces were stained with the antibodies listed in the supplement at 4°C for 30 min. Data were acquired with a BD FACSCanto II system and analyzed with FlowJo software (Tree Star, Inc.).

Antibody for flow cytometry

| Antibody | Reagent Plant | Cat.No. |
| --- | --- | --- |
| Tregs | | |
| CD4-PE | Biolegend | 317410 |
| CD25-PE | Biolegend | 302606 |
| CD127-APC | Biolegend | 351316 |
| CD3-4-8, PD-1 | | |
| CD3-PE-cy7 | Biolegend | 344816 |
| CD4-PE | Biolegend | 317410 |
| CD8-FITC | Biolegend | 301050 |
| PD-1-APC | Biolegend | 329908 |
| CD45-PERCP-CY5.5 | BD | 340953 |
| NK, B absolute counter | | |
| CD3-FITC | Biolegend | 300306 |
| CD56-PE | Biolegend | 362508 |
| CD16-PE | Biolegend | 360704 |
| CD19-APC | Biolegend | 302212 |
| CD45-PERCP-CY5.5 | BD | 340953 |
| Absolute counter tube | BD | 340334 |

##

## Fig. S1 Comparison of baseline PBIC profiles between the TRG0 and non-TRG0 groups.


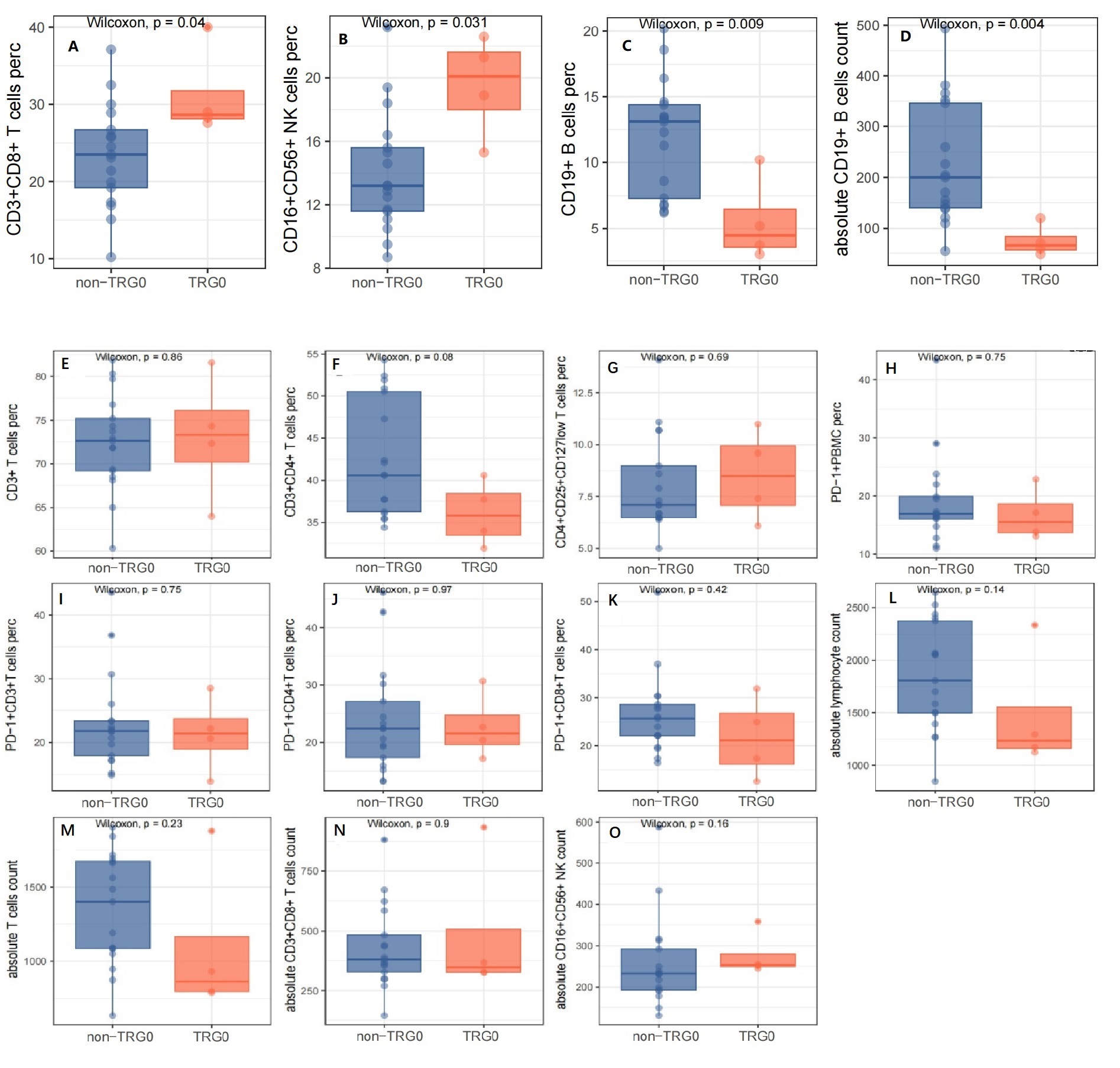


(a-d) Significant differences in PBIC profiles of baseline between the TRG0 and non-TRG0 groups. Differeces of CD3+CD8+ T cells, CD16+CD56+ NK cells, CD19+ B cells, and the absolute counts of CD19+ B cells in pretreatment PBIC profiles between non-TRG0 and TRG0 group.(e) Box plots were used to visualize the differences in CD3+ T cells between the TRG0 and non-TRG0 groups. (f) Differential analysis of CD3+CD4+ T cells between TRG0 and non-TRG0 groups. (g) Differential analysis of CD4+CD25+CD127low T cells between TRG0 and non-TRG0 groups. (h) Differential analysis of PD-1+PBMC between TRG0 and non-TRG0 groups. (i) Differential analysis of PD-1+CD3+ T cell between TRG0 and non-TRG0 groups. (j) Differential analysis of PD-1+CD4+ T cell between TRG0 and non-TRG0 groups. (k) Differential analysis of PD-1+CD8+ T cell between TRG0 and non-TRG0 groups. (l) Differential analysis of absolute lymphocyte count between TRG0 and non-TRG0 groups. (m) Differential analysis of absolute T cells counts between TRG0 and non-TRG0 groups. (n) Differential analysis of absolute CD3+CD8+ T cells count between TRG0 and non-TRG0 groups. (o) Differential analysis of absolute CD16+ CD56+ NK cells count between TRG0 and non-TRG0 groups.

## Fig. S2 Comparison of PBIC profiles before and after surgery in the TRG0 and non-TRG0 groups.


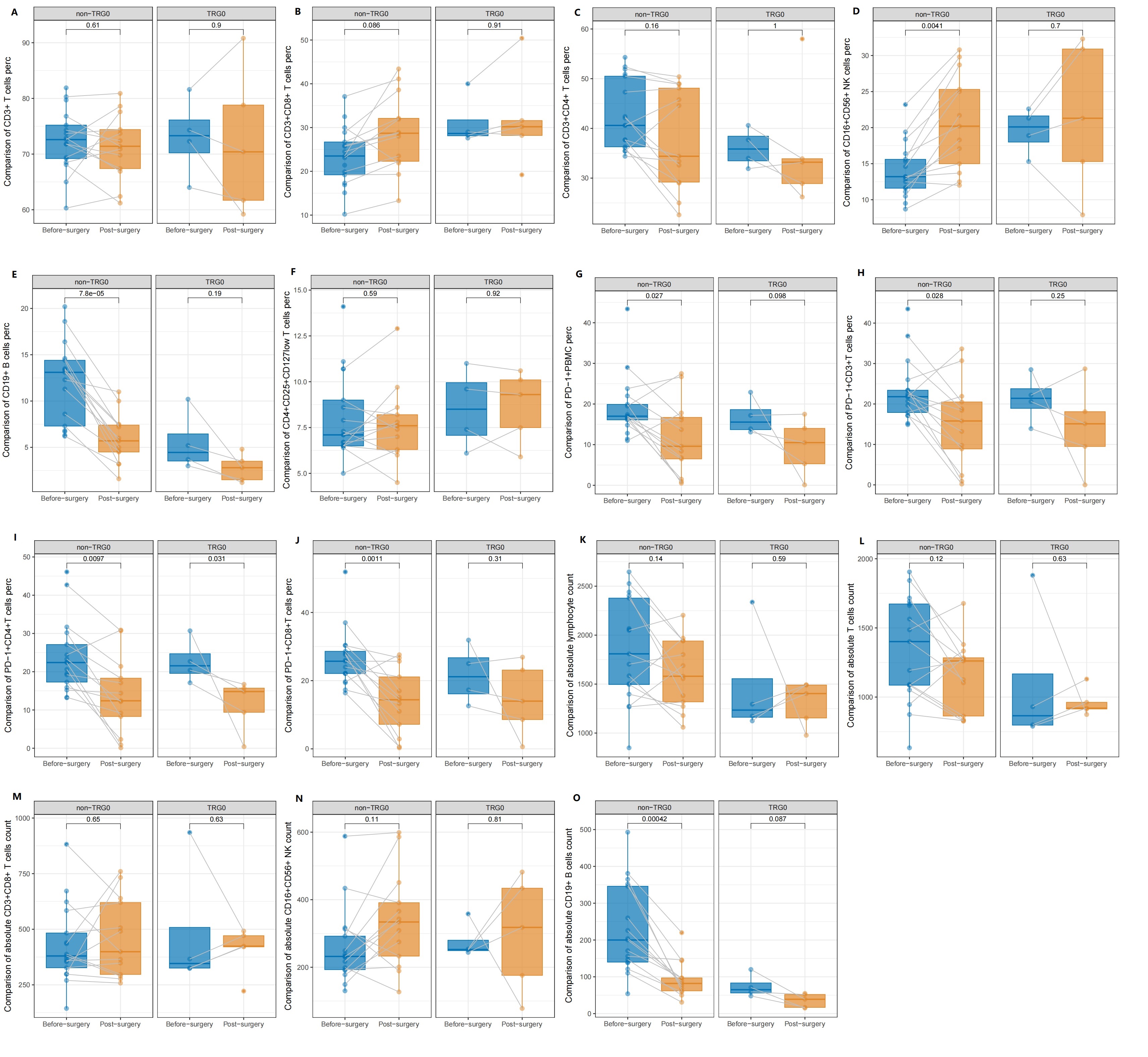


(a) Box plots of CD3+ T cell levels before and after surgery (non-TRG0 and TRG0 groups). (b) Box plots of CD3+CD8+ T cell levels before and after surgery (non-TRG0 and TRG0 groups). (c) Box plots of CD3+CD4+ T cell levels before and after surgery (non-TRG0 and TRG0 groups). (d) Box plots of CD16+CD56+ NK cell levels before and after surgery (non-TRG0 and TRG0 groups). (e) Box plots of CD19+ B cell levels before and after surgery (non-TRG0 and TRG0 groups). (f) Box plots of CD4+CD25+CD127low T cell levels before and after surgery (non-TRG0 and TRG0 groups). (g) Box plots of PD-1+ PBMC levels before and after surgery (non-TRG0 and TRG0 groups). (h) Box plots of PD-1+CD3+ T cell levels before and after surgery (non-TRG0 and TRG0 groups). (i) Box plots of PD-1+CD4+ T cell levels before and after surgery (non-TRG0 and TRG0 groups). (j) Box plots of PD-1+CD8+ T cell levels before and after surgery (non-TRG0 and TRG0 groups). (k) Comparison of absolute lymphocyte count before and after surgery (non-TRG0 and TRG0 groups). (l) Comparison of absolute T cell count before and after surgery (non-TRG0 and TRG0 groups). (m) Comparison of absolute CD3+CD8+ T cell count before and after surgery (non-TRG0 and TRG0 groups). (n) Comparison of absolute CD16+CD56+ NK cell count before and after surgery (non-TRG0 and TRG0 groups). (o) Comparison of absolute CD19+ B cell count before and after surgery (non-TRG0 and TRG0 groups).

## Table S1. Cycles of neoadjuvant (n=25) and adjuvant treatment (n=20)

|  | **Patients** |
| --- | --- |
| Neoadjuvant camrelizumab* |  |
| 3 cycles | 24/25 (96.0) |
| 2 cycles | 1/25 (4.0) |
| Neoadjuvant trastuzumab |  |
| 5 cycles | 1/25 (4.0) |
| 4 cycles | 20/25 (80.0) |
| 3 cycles | 4/25 (16.0) |
| Neoadjuvant CapOx |  |
| 5 cycles** | 3/25 (12.0) |
| 4 cycles | 19/25 (76.0) |
| 3 cycles | 3/25 (12.0) |
| Adjuvant CapOx |  |
| 4 cycles | 14/20 (70.0) |
| 3 cycles | 3/20 (15.0) |
| 2 cycles | 2/20 (10.0) |
| 1 cycle# | 1/20 (5.0) |

Data are median (range) or n (%). *24 patients were prescribed 3 cycles of camrelizumab. **Three patients insisted on receiving 5 cycles of neoadjuvant therapy before undergoing surgery, with only CapOx chemotherapy administered in the fifth cycle. #One patient refused chemotherapy due to personal willingness after receiving one cycle of adjuvant CapOx.

## Table S2. Subgroup analysis of pCR after neoadjuvant treatment (n=23)

|  | Events/Patients |
| --- | --- |
| Age |  |
| ≤65 | 4/15 (26.7) |
| >65 | 1/8 (12.5) |
| Gender |  |
| Female | 0/5 (0.0) |
| Male | 5/18 (27.8) |
| Primary tumor location |  |
| Gastric | 3/17 (17.6) |
| Gastric-esophageal junction | 2/6 (33.3) |
| Histologic grade |  |
| Moderately to highly differentiated | 1/11 9.1) |
| Poorly differentiated | 1/5 (20.0) |
| Unknown | 3/7 (42.9) |
| Microsatellite status |  |
| MSS | 4/22 (18.2) |
| Unavailable | 1/1 (100.0) |
| PD-L1 (CPS) |  |
| <1 | 2/12 (16.7) |
| ≥1 | 3/11 (27.3) |

Data are n (%). MSS, microsatellite stable; PD-L1, programmed death-ligand 1; CPS, combined positive score.

## Table S3. Surgical outcomes in the surgery set (n=23)

|  | **Patients** |
| --- | --- |
| Time interval from the last neoadjuvant treatment to surgery, day | 33 (24, 85) |
| Time interval from the completion of surgery to the initiation of adjuvant treatment, day | 31 (24, 47) |
| Number of lymph nodes cleared, mean (range) | 27 (14, 46) |
| Operative duration, min | 250 (180, 335) |
| Intraoperative blood loss, mL | 125 (30, 500) |
| Postoperative hospital stay, day | 11 (5, 16) |
| Postoperative 90-day mortality | 0 |
| Secondary surgery | 0 |

Data are median (range).

## Table S4. Immune-related adverse events during neoadjuvant treatment (n=25)

| irAEs | Any grade | Grade 1 | Grade 2 | Grade 3 |
| --- | --- | --- | --- | --- |
| RCCEP | 10 (40.0) | 9 (36.0) | 1 (4.0) | 0 |
| CPK increased | 7 (28.0) | 6 (24.0) | 0 | 1 (4.0) |
| Pruritus | 2 (8.0) | 2 (8.0) | 0 | 0 |
| Hypothyroidism | 2 (8.0) | 1 (4.0) | 1 (4.0) | 0 |
| Hyperthyroidism | 1 (4.0) | 1 (4.0) | 0 | 0 |
| Increased creatinine | 1 (4.0) | 1 (4.0) | 0 | 0 |
| Diarrhea | 1 (4.0) | 0 | 0 | 1 (4.0) |

Data are n (%). No ≥grade 4 irAEs occurred. irAEs, immune-related adverse events; RCCEP, reactive cutaneous capillary endothelial proliferation; CPK, creatine phosphokinase.

## Table S5. Surgical complications in the surgery set (n=23)

| Surgical complications | Grade I-II | Grade ≥ III |
| --- | --- | --- |
| Nausea | 12 (52.2) | 0 |
| Fatigue | 11 (47.8) | 0 |
| AST increased | 9 (39.1) | 0 |
| ALT increased | 8 (34.8) | 0 |
| Anemia | 8 (34.8) | 0 |
| Hypoproteinemia | 8 (34.8) | 0 |
| Fever | 7 (26.1) | 0 |
| Vomiting | 5 (21.7) | 0 |
| Pneumonia | 2 (8.7) | 0 |
| Bleeding | 1 (4.3) | 0 |
| Chylous leakage | 1 (4.3) | 0 |

Data are n (%). ALT, alanine aminotransferase; AST, aspartate aminotransferase.

## Table S6. Treatment-emergent adverse events during adjuvant treatment (n=20)

| TEAEs | Any grade | Grade 1 | Grade 2 | Grade 3 |
| --- | --- | --- | --- | --- |
| Lymphopenia | 15 (75.0) | 8 (40.0) | 1 (5.0) | 6 (30.0) |
| Nausea | 15 (75.0) | 12 (60.0) | 3 (15) | 0 |
| Fatigue | 13 (65.0) | 13 (65.0) | 0 | 0 |
| Platelet count decrease | 12 (60.0) | 2 (10.0) | 8 (40.0) | 2 (10.0) |
| Anorexia | 12 (60.0) | 11 (55.0) | 1 (5.0) | 0 |
| Neutropenia | 12 (60.0) | 5 (25.0) | 5 (25.0) | 2 (10.0) |
| Leukopenia | 11 (55.0) | 5 (25.0) | 6 (30.0) | 0 |
| Hypoproteinemia | 11 (55.0) | 10 (50.0) | 1 (5.0) | 0 |
| Vomiting | 10 (50.0) | 8 (40.0) | 2 (10.0) | 0 |
| Anemia | 10 (50.0) | 7 (35.0) | 3 (15.0) | 0 |
| Creatine phosphokinase increase | 5 (25.0) | 5 (25.0) | 0 | 0 |
| Direct bilirubin increase | 4 (20.0) | 2 (10.0) | 2 (10.0) | 0 |
| Aspartate aminotransferase increase | 4 (20.0) | 4 (20.0) | 0 | 0 |
| Indirect bilirubin increase | 4 (20.0) | 4 (20.0) | 0 | 0 |
| Alkaline phosphatase increase | 2 (10.0) | 1 (5.0) | 1 (5.0) | 0 |
| Alanine aminotransferase increase | 2 (10.0) | 2 (10.0) | 0 | 0 |
| Total bilirubin | 2 (10.0) | 1 (5.0) | 1 (5.0) | 0 |
| Allergy | 2 (10.0) | 2 (10.0) | 0 | 0 |
| Hypothyroidism | 1 (5.0) | 0 | 1 (5.0) | 0 |
| Fever | 1 (5.0) | 1 (5.0) | 0 | 0 |
| Increased creatinine | 1 (5.0) | 1 (5.0) | 0 | 0 |

Data are n (%). No ≥grade 4 TEAEs occurred. TEAEs, treatment-emergent adverse events.
